# A Fabrication Method for Realizing Vertically Aligned Silicon Nanowires Featuring Precise Dimension Control

**DOI:** 10.3390/s24227144

**Published:** 2024-11-06

**Authors:** Sourav Mukherjee, Mohannad Y. Elsayed, Hani H. Tawfik, Mourad N. El-Gamal

**Affiliations:** 1 Electrical and Computer Engineering, McGill University, Montreal, QC H3A 0E9, Canada; mourad.el-gamal@mcgill.ca; 2 MEMS Vision International Inc., Montreal, QC H4P 2R9, Canada; mohannad.elsayed@mems-vision.com (M.Y.E.); hani.tawfik@mems-vision.com (H.H.T.)

**Keywords:** silicon nanowire, e-beam lithography, EBL, dry etching, DRIE, nanofabrication, nanostructures, Bosch process

## Abstract

Silicon nanowires (SiNWs) have garnered considerable attention in the last few decades owing to their versatile applications. One extremely desirable aspect of fabricating SiNWs is controlling their dimensions and alignment. In addition, strict control of surface roughness or diameter modulation is another key parameter for enhanced performance in applications such as photovoltaics, thermoelectric devices, etc. This study investigates a method of fabricating silicon nanowires using electron beam lithography (EBL) and the deep reactive ion etching (DRIE) Bosch process to achieve precisely controlled fabrication. The fabricated nanowires had a pitch error within 2% of the pitch of the direct writing mask. The maximum error in the average diameter was close to 25%. The simplified two-step method with tight control of the dimensions and surface tunability presents a reliable technique to fabricate vertically aligned SiNWs for some targeted applications.

## 1. Introduction

With the advent of advanced modern nanofabrication techniques, new nanostructures and their applications have been extensively explored. Over the last few decades, a major focus has been geared towards silicon nanowires (SiNWs). SiNWs display incredible electrical, mechanical, optical, and thermal properties, making them a major focus in the investigation of different applications. As the world is desperately looking to find beyond-Moore innovations that can replace the saturating CMOS technology, nanostructures such as SiNWs ignite special interest. Numerous studies have been conducted on silicon nanowire-based transistors and microarchitectures [1,2,3,4]. Another important area of application of SiNWs is photovoltaics. SiNW-based solar cells yield more efficient light absorption owing to their large surface-area-to-volume ratios and low reflections [5,6]. SiNWs have also been extensively used to build novel sensors with high sensitivity and small form factors. Sensory applications such as gas sensing and less invasive blood glucose detection are some, to name a few [7,8]. Extremely small diameter nanowires also exhibit quantum confinement, a phenomenon that results in widening of the energy bandgap, which has attracted much interest as well [9]. The new generation of batteries also exploits SiNW-based anodes, which result in extremely promising performances. This technology is already pushing the electric vehicle (EV) revolution through the commercialization of SiNW batteries that can provide an unprecedented energy density of 500 Wh/kg [10]. Needless to say, there have been continuous efforts to use nanowires in new non-traditional applications.

Controlling the dimensions of silicon nanowires is extremely desirable in nanotechnology; however, achieving this is not straightforward. In addition, different applications can require higher control over the alignment [11]. There are primarily two approaches to fabricate silicon nanowires: bottom-up and top-down approaches.

The most common bottom-up fabrication approach is the use of vapor–liquid–solid (VLS) growth to synthesize nanowires. The method requires the deposition of one metal catalyst (Au, Al, etc.), often using sputtering or electron beam evaporation, which determines the diameter, density, and alignment of the nanowires [12,13,14]. The reliance on the catalyst parameters often results in loosely controlled fabrication in terms of the nanowire dimensions. Controlling the alignment of the fabricated SiNWs is also challenging to control. SiNWs often bend and entangle with each other, and controlling their pitch is challenging.

Metal-assisted chemical etching (MACE or MACEtch) is a popular top-down method for fabricating silicon nanowires because it is inexpensive and less complex. In the first step, the substrate needs to be covered with metal catalyst nanoparticles, either by depositing a thin film using evaporation/sputtering or by electroless deposition. The substrate is then etched in an HF and H_2_O_2_-based solution to obtain vertical nanowires [15,16,17]. The process requires the metal film to be porous, as the area under the metal catalyst is etched away, and the un-patterned or holey area withstands the etching process, resulting in freestanding SiNWs. Again, the random nature of the nanoparticle deposition causes uncontrolled nanowire growth. The process is also crystal-orientation-dependent, as the orientation dictates the growth direction of the nanowires. One way to increase controllability is to use nanospheres to define the nanowire diameter [18]. This process increases the control of the nanowire diameter; however, achieving the required density and spacing remains a challenge, primarily because of the inability to control the deposition of nanospheres and metal thin film porosity. In [19], the authors used a slightly modified approach in which they used electron beam lithography (EBL) to define the metal mask, followed by MACEtch (etch) of the exposed area to produce nanowires. The authors mentioned the existence of nanogaps around the nanowires, which can change the etching process. Moreover, the thickness and porosity of the thin film still remain key factors for the successful implementation of the process. There have been many studies on fabricating horizontal silicon nanowires using electron beam lithography [20,21], but vertically aligned silicon nanowire fabrication is mostly dominated by MACE, which has several issues, as discussed earlier.

In this study, we propose a method that combines EBL and deep reactive ion etching (DRIE) to fabricate highly controlled SiNWs. The direct writing process eliminates the requirement of a physical mask (which often incurs additional costs and delays the process), whereas the DRIE process provides a vertical anisotropic etching profile for the nanowires. The flexibility in the digital mask design results in an easily tunable nanowire shape.

First, the fabrication process flow is explained, and then the measurements and analysis are discussed, followed by conclusions.

## 2. Materials and Methods

**Figure 1:**
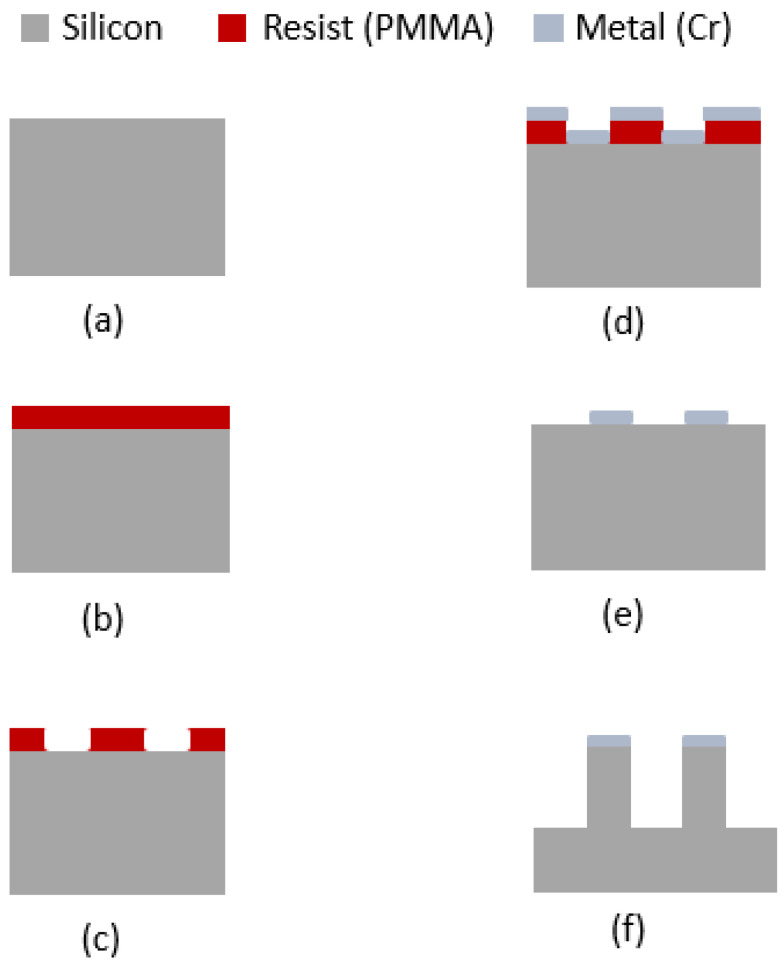
Fabrication process flow: (**a**) HF-cleaned silicon substate, (**b**) spin coating with PMMA, (**c**) patterning using EBL, (**d**) metal (Cr) thin film deposited using electron beam evaporation, (**e**) metal (Cr) mask after lift-off, and (**f**) free-standing vertical nanowires after the etching process (scalloping not shown).

We used a p-type <100> single-crystal silicon substrate with a resistivity of 0.003–0.005 Ω·cm. EBL was used to create a mask to control the fabrication of the SiNWs, and DRIE was used to etch the bulk silicon. The complete fabrication process is illustrated in Figure 1.

At first, the sample was dipped into HF to remove the native oxide and rinsed with DI water, as shown in Figure 1a. The sample was then dried and pre-baked at 180 °C for 5 min on a hotplate. After the sample cooled down, spin coating was employed to deposit a resist on the silicon substrate. We opted for positive EBL resist poly(methyl methacrylate) (PMMA) 950K A4 (Microchem) to coat the sample using a Laurell WS-400B-6NPP-LITE spin coater. With the spin parameters shown in Table 1, a resist thickness of close to 200 nm was achieved (verified using a profilometer), as shown in Figure 1b. The sample was then hotplate-baked again at 180 °C for 90 s.

The sample was subsequently processed with EBL. The purpose of this study is to investigate the controllability of silicon nanowire growth for nanowires of different diameters and pitches, especially for applications such as solar cells, thermoelectric devices, ionization gas sensors, etc. Although these designs can widely vary based on the specific application and objective, sub-micron designs of a few hundred nanometers are very popular. The EBL writing was thus divided into three groups to characterize the nanowire density and diameter, as follows:

(a) Group 1: Matrix of 200 nm diameter circles with 200 nm inter-nanowire spacing.
(b) Group 2: Matrix of 400 nm diameter circles with 400 nm inter-nanowire spacing.
(c) Group 3: Matrix of 600 nm diameter circles with 600 nm inter-nanowire spacing.

A Raith e-line EBL/SEM machine was used for direct writing. The EBL parameters are listed in Table 2. Among the parameters listed, dose was found to be the most important factor. The choice of energy dose can significantly impact the exposure process and resolution of the nanostructures. Additionally, the dose must be calibrated for each geometry.

After exposure, development was performed using a solution of MIBK:IPA (1:3) for 60 s. The sample was subsequently rinsed with isopropyl alcohol (IPA) for 15 s and finally dried using nitrogen. The development process creates circular holes in the PMMA film, as shown in Figure 1c.

After development, a 50 nm layer of chromium was then deposited (Figure 1d) using a BJD 1800 e-beam evaporator tool manufactured by Temescal. Thereafter, a metal lift-off was performed in acetone (Figure 1e) with agitation. The lift-off process completely removes all PMMA and produces the chromium metal mask. The metal thickness was selected to be enough as a hard mask for the subsequent DRIE etching of the nanowires up to the desired length. The PMMA thickness was set to be close to four times the metal thickness to facilitate a smooth lift-off process.

After the chromium (Cr) mask was created, the exposed silicon sample was etched using the DRIE Bosch process as shown in Table 3. The process requires SF_6_ and C_4_F_8_ gases, where SF_6_ is the etchant and C_4_F_8_ is used for passivation. In this cyclical method, the etching and passivation steps are alternated. A buffer step has been introduced between the etching and passivation phases to facilitate a smooth transition. Diameter modulation or scalloping size tuning, which is a primary objective for the applications we are targeting, can be executed by tuning the etching/passivation periods and the flow rate. We performed the DRIE in a Plasmalab System 100 model manufactured by Oxford Instruments.

There have been previous efforts to fabricate silicon nanowires using traditional reactive ion etching (RIE), but they were lacking in several aspects [22]. The structure generated by RIE often lacks the directionality required to implement vertical structures. Most importantly, reaching greater depths may be a challenge with RIE, as opposed to DRIE. Some applications of silicon nanowires require the nanostructures to have a smooth sidewall. There have been several papers often dealing with this challenge, where the nanowires are fabricated using cryogenic DRIE or pseudo-Bosch processes [23,24]. On the other hand, some applications require a customizable sidewall profile. This is where our technique fits in.

In SiNW-based solar cell fabrication, both nanowire height and diameter impact the light-trapping capability, and deviation in the dimensions can result in considerable changes in the expected results. The authors in [25] analyzed the impact of nanowire diameter and height, showing that certain optimal dimensions exist. In that study, the authors fabricated nanowire diameters ranging from 200 nm to 900 nm. They found that 200 nm diameter nanowires showed the lowest reflectivity, whereas 600 nm diameter nanowires resulted in the best photovoltaic conversion efficiency (PCE). In [26], the impact of the inter-wire spacing was also examined, which showed for a certain diameter an optimal spacing resulting in the highest photocurrent. Hence, tight control on the dimensions is extremely desirable. Apart from the dimension and pitch control, the surface morphology is another important aspect. For example, in [27], a significant reduction in thermal conductivity was observed for nanowires with scalloping, exhibiting far better performance compared to the SiNWs realized using MACE. A thermal conductivity of 60% less than the Casimir limit was achieved for 200 nm nanowires with a scalloping period of 55 nm. Such results are extremely encouraging for thermoelectric devices. Surface waviness has proved to be helpful in improving solar cell performances as well. In [28], the authors showed that nanowires with a tuned diameter could be a better choice for higher optical absorption than cylindrical nanowires. The authors performed a finite difference time domain (FDTD) simulation on a 410 nm cylindrical (smooth surface) nanowire and a diameter modulated nanowire where the period was 565 nm and modulated diameters were 495 and 380 nm at the convex and concave points, respectively. The simulation showed strong, closely spaced resonance peaks, performing far better than the cylindrical nanowire. They verified 23.4% higher absorption for the diameter-modulated nanowire. The authors used photolithography and DRIE to fabricate the nanowires. The process lacks direct control over the final diameter, as the photolithography process in an academic setting generally prevents reaching nanoscopic resolution, hence requiring an additional thermal treatment to reduce the diameter using oxidation. With such a process, controlled fabrication of smaller diameter nanowires would be hard to realize. Another application is the development of micro-ionization gas sensors (IGS), where the surface roughness obtained during the Bosch process is used to facilitate low-voltage ionization [29]. The rough surface can be used as the ionizing emitter for such applications. In microfluidic applications, greater flow control is desirable, which can be obtained by the anisotropic wetting of the surface. Implementing scalloped nanogrooves (SNGs) has proven to be a known method to achieve that [30].

The discussion so far makes it very obvious that we need an etching method that not only provides tight control but also gives the liberty to tune the surface roughness or the waviness of the wire. No other prominent nanofabrication technique except the traditional Bosch process is capable of satisfying our requirements of controllably modulating the nanowire sidewall during the etching process for such applications. Hence, this paper focused on fabricating nanowires using a Bosch DRIE process.

## 3. Characterization and Results

**Figure 2:**
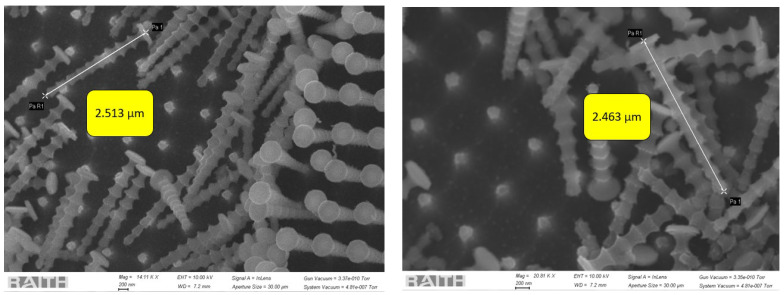
Height of the nanowires (Group 2).

To characterize the fabricated nanowires, we used the same EBL tool (at an accelerating voltage of 10 kV). Some part of Group 2 was intentionally scratched to obtain a good view of the sidewalls of the nanowires. Figure 2 indicates that the height of the nanowires in this region is ~2.5 µm (an aspect ratio of 6). As all three groups were present in the same sample and were processed by the same process at the same time, the height in the other groups should be close to what we obtained in Group 2.

We also examined the fabricated nanowires and checked whether they met the expected dimensions based on our EBL writing. We investigated the fabricated diameter, pitch, and also the alignment.

**Figure 3:**
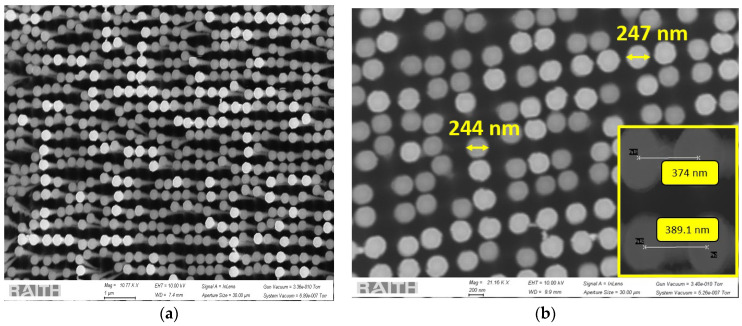
SEM images of the silicon nanowires in Group 1 with (**a**) a 3D view and (**b**) diameter and pitch (in inset) from the top view.

Figure 3a shows a tilted top view of the fabricated silicon nanowire array in Group 1. This group had the smallest diameter and the least spacing between the nanowires, resulting in a denser jungle of nanowires. Figure 3b shows the nanowire diameter, and the inset shows the pitch. The SiNWs had some Cr remaining on their tips.

**Figure 4:**
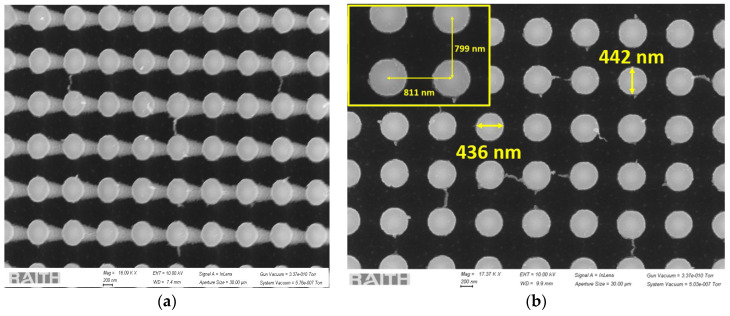
SEM images of the silicon nanowires in Group 2 with (**a**) a 3D view and (**b**) diameter and pitch (in inset) from the top view.

The SEM images of Group 2 are shown in Figure 4. The three-dimensional view in Figure 4a shows highly aligned vertical nanowire growth. The nanowires were evenly separated and well aligned. The metal mask clearly remains intact and can be removed using a Cr etchant if needed.

**Figure 5:**
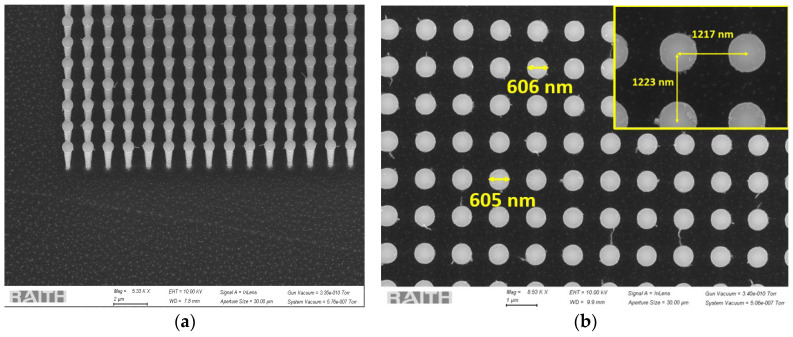
SEM images of the silicon nanowires in Group 3 with (**a**) a 3D view and (**b**) diameter and pitch (in inset) from the top view.

Similar to Group 2, Group 3 also exhibited extreme control and vertical alignment of the nanowires, as shown in Figure 5. Such anisotropic etching would be difficult to achieve with RIE.

All the SEM images of these three different groups, as shown in Figure 3, Figure 4 and Figure 5, with measurements of the nanowire diameters and pitch, also confirm the proper vertical alignment of the nanowires. The scalloping effect resulting from the Bosch process is clearly evident in Figure 2, Figure 4a and Figure 5a. The EBL process is used for strict dimension control at the nanoscopic level, whereas the Bosch DRIE has been implemented in order to have an anisotropic and customized sidewall profile. Although having a scalloped sidewall is beneficial for our targeted applications, a smoother sidewall is still possible using the proposed method. One common way is by optimizing DRIE parameters, such as reducing the C_4_F_8_ flow rate, increasing the switching speed, optimizing chamber pressure, etc. Alternatively, a significant smoothing of the sidewall is possible through repeated thermal treatments (oxidation and SiO_2_ removal) [31].

To analyze the effectiveness of the method to improve the performance of specific applications, we performed an FDTD simulation on cylindrical and scalloped nanowires. The unit cell was 0.5 µm in length and width, whereas the computational height was chosen to be 4.5 µm. The cell unit consisted of a single nanowire having a periodic condition in the x and y planes, and the z direction was assumed to have a perfectly matched layer (PML). An incident plane wave generated from a source placed above the nanowire illuminated the silicon nanowire in the cell. Two power monitors were used to measure the reflection and transmission, from which we calculated the absorption value. We assessed three cases (all having the same height of 2.5 µm): a cylindrical nanowire and two scalloped nanowires. The scalloping was modeled using a sinusoidal function having a modulation between 400 nm (at convex point) and 250 nm (at concave point). The modulation period is defined as the distance between two adjacent convex or concave points. In our scalloped nanowires, only the modulation period changed (250 nm for profile 1 and 1000 nm for profile 2) for these two profiles. The scalloped nanowire in profile 1 exactly mimics our nanowires in Group 2, whereas the nanowires in profile 2 are easily producible by simply changing the etch/passivation time.

**Figure 6:**
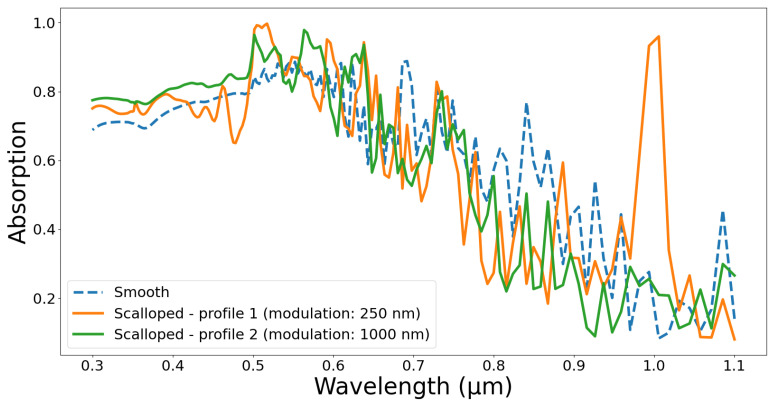
Absorption spectra of cylindrical (diameter = 400 nm, height = 2.5 µm) and scalloped nanowires (profile 1: diameter = 400 nm at convex points and 250 nm at concave points, modulation period = 250 nm; profile 2: diameter = 400 nm at convex points and 250 nm at concave points, modulation period = 1000 nm).

Figure 6 shows the absorption spectra of the cylindrical and scalloped silicon nanowires. It can be observed that the scalloped nanowires resulted in higher and wider peaks due to the resonance caused by the surface modulation. High absorption is especially important for solar cell and photodetector applications. Apart from the variation in modulation period, the diameter, pitch, and other factors also impact absorption, which is beyond the scope of the study.

## 4. Analysis

We analyzed the nanowire dimensions (diameter and spacing/pitch) using ImageJ software (version 1.54g). Table 4 shows that we can rely on the presented process to fabricate nanowires in a more controllable manner.

All three groups showed extreme predictability of the nanowire pitch, with a fabrication error of less than two percent. This extraordinary precision can be leveraged in areas where pitch is of utmost importance.

In terms of nanowire diameter, Group 3 showed the best predictability, with less than four percent deviation from the mask patterns. In Group 1, the nanowires with the least pitch (400 nm pitch at mask level) and smallest diameter (200 nm) showed the highest error in the average diameter, which was close to 25%. Due to the batch processing, all three groups experienced the same processing, which resulted in suboptimal conditioning in some regions. In a practical scenario where the application targets a particular geometry, the deviation can easily be reduced by improving the EBL resolution (by optimizing the area doses, focus, etc.) and lift-off. Moreover, the DRIE parameters can be tuned for scalloping control and undercut optimization.

Table 5 compares our method with other popular DRIE methods used in the literature, showcasing the novelty of the work. By combining EBL with a Bosch DRIE process, the simplified two-step method yields tight control over the pitch and diameter of SiNWs without the need for any additional steps. While other techniques are lacking, this method offers high tunability of the scalloping, which is critical to optimizing device performance in applications discussed before. In addition, room-temperature operation eliminates any need for costly cooling requirements as seen in cryogenic DRIE methods, making our process a cost-effective and scalable method for a wide range of applications.

## 5. Conclusions

In this study, a highly controllable method for fabricating silicon nanowires was presented. The method is based on EBL and DRIE to provide superior control over the nanowire diameter, pitch, length, and alignment. The fabricated nanowires had a pitch error within 2% of the pitch of the direct writing mask. The maximum error in the average diameter was 25% of the EBL mask. The controllability of the dimensions and alignment can be utilized in several previously mentioned applications. Additionally, the controllability of the surface roughness and diameter modulation makes the process tuned for applications such as solar cells, IGS, thermoelectric devices, etc. The process proposed here could be a starting point for fabricating customized solutions for such applications.

## Figures and Tables

**Table 1:** Spin coating parameters.

Parameter	Value
Spread	500 rpm, 5 s
Acceleration	1305 rpm/s
Spin	5000 rpm, 30 s
Deceleration	5 s

**Table 2:** EBL direct writing parameters.

Parameter	Value
Nanopatterns	Circles
Acceleration voltage	10 kV
Dose	100 µC/cm^2^
Aperture	30 µm
Working distance	10 mm

**Table 3:** DRIE key process parameters.

Process Parameters	Passivation Step	Transition (Buffer) Step	Etching Step
C_4_F_8_ flow rate (sccm)	65	15	15
SF_6_ flow rate (sccm)	1	65	65
Chamber pressure (mTorr)	20	20	20
Stage temperature (°C)	20	20	20
ICP power (W)	450	0	450
CCP power (W)	10	0	25
He cooling (Torr)	11	11	11
Time (s)	3	2	7

**Table 4:** Fabricated device analysis.

Parameters	Group 1	Group 2	Group 3
Diameter (mask)	200 nm	400 nm	600 nm
Average diameter (fabricated)	251.6 nm	445 nm	623.9 nm
Average diameter (error)	~25.8%	~11.25%	~4%
Average pitch (mask)	400 nm	800 nm	1200 nm
Average pitch (fabricated)	403.1 nm	816 nm	1221.7 nm
Average pitch (error)	~0.77%	~2%	~1.8%

**Table 5:** Comparison with other common DRIE-based methods.

Metric	Our Method (EBL + Bosch DRIE)	Photolithography + Bosch DRIE [28]	EBL + Cryogenic DRIE [24]	EBL + Pseudo-Bosch DRIE [32,33]
Pitch control	Very high	-	-	-
Diameter control	High	Moderate	High	Moderate
Additional treatment	No	Yes (thermal oxidation and oxide etching)	No	No
Sidewall tunability	Very high	Very high	Low	Low
Cooling requirement	Low—Standard temperature conditions required	Low—Standard temperature conditions required	Very high—Cryogenic cooling is required	Moderate—Requires some cooling but less stringent than cryo-DRIE
Target applications	Thermoelectric devices, IGS, solar cells, etc.	Solar cells, photodetectors, etc.	High-end electronics, optoelectronics, sensors requiring smooth surfaces	MEMS, high aspect ratio structures

## Data Availability

Data are contained within the article.
